# Phagocytic ability declines with age in adult Drosophila hemocytes

**DOI:** 10.1111/acel.12227

**Published:** 2014-05-14

**Authors:** Lucas Horn, Jeff Leips, Michelle Starz-Gaiano

**Affiliations:** Department of Biological Sciences, University of Maryland Baltimore CountyBaltimore, MD, 21250, USA

**Keywords:** Drosophila, hemocytes, immunity, immunosenescence, phagocytosis, senescence

## Abstract

Most multicellular organisms show a physiological decline in immune function with age. However, little is known about the mechanisms underlying these changes. We examined *Drosophila melanogaster,* an important model for identifying genes affecting innate immunity and senescence, to explore the role of phagocytosis in age-related immune dysfunction. We characterized the localized response of immune cells at the dorsal vessel to bacterial infection in 1-week- and 5-week-old flies. We developed a quantitative phagocytosis assay for adult Drosophila and utilized this to characterize the effect of age on phagocytosis in transgenic and natural variant lines. We showed that genes necessary for bacterial engulfment in other contexts are also required in adult flies. We found that blood cells from young and old flies initially engulf bacteria equally well, while cells from older flies accumulate phagocytic vesicles and thus are less capable of destroying pathogens. Our results have broad implications for understanding how the breakdown in cellular processes influences immune function with age.

## Introduction

Age-related decline in immune function, or immunosenescence, appears to be a general hallmark of aging in multicellular organisms. For humans, this decline poses a serious health risk and commonly leads to hospitalization in the elderly (High, 2004; Ongradi & Kovesdi, 2010). Although changes in adaptive immunity were once thought to be the primary cause of immunosenescence, recent evidence demonstrates that age-related decline in functional components of the innate immune system also plays a significant role (Plowden *et al*., 2004; Panda *et al*., 2009).

To examine the key factors in age-related functional decline of innate immunity, we employed a genetic model organism, the fruit fly, *Drosophila melanogaster*. Drosophila and humans share a similar response to bodily infections, particularly in the molecular pathways of the innate immune response (Lemaitre & Hoffmann, 2007). With only an innate immune system, studies in Drosophila can focus on the consequences of age-related changes in innate immunity without the complicating interactions arising from adaptive immune components.

Adult Drosophila respond to infection in two ways: clearance of pathogens by phagocytic hemocytes (also called blood cells or plasmatocytes) and production of antimicrobial proteins (AMPs) [reviewed in (Lemaitre & Hoffmann, 2007)]. Phagocytosis begins in minutes in response to bacteria that have breached the cuticle or digestive tract, and is required for survival of an infection (Nehme *et al*., 2007; Stuart & Ezekowitz, 2008; Charroux & Royet, 2009). This cellular response has been best characterized in embryos, larvae, and in cell culture assays [reviewed in (Stuart & Ezekowitz, 2008; Fauvarque & Williams, 2011; Ulvila *et al*., 2011)], but a few qualitative studies have been carried out in adult flies (Elrod-Erickson *et al*., 2000; Kocks *et al*., 2005; Garver *et al*., 2006; Mackenzie *et al*., 2011; Nehme *et al*., 2011). Bacteria that are not immediately engulfed can be destroyed by AMPs, which are released by various cells (Lemaitre & Hoffmann, 2007; Stuart & Ezekowitz, 2008). However, transcriptionally regulated AMP production is delayed compared with phagocytosis (Ramet *et al*., 2002; Lemaitre & Hoffmann, 2007; Haine *et al*., 2008). While decline in either or both of these components could contribute to immunosenescence, their individual roles have not been characterized in most studies because common assays, like the ability to survive and/or clear bacterial infection, combine the effects of both.

Research in a variety of organisms suggests a model for how phagocytic cells destroy bacteria [reviewed in (Nordenfelt & Tapper, 2011)]. Contact with a pathogen activates cell-surface receptors on hemocytes, triggering cytoskeletal changes and the formation of a phagocytic cup that develops into a membrane-bound phagosome containing the invading pathogen(s). Phagosomes fuse with lysosomes to create phagolysosomes, and the low pH in these compartments destroys the bacteria. Only some of the molecules required for this cellular process are known.

Mutant analyses in Drosophila have identified several genes required for efficient bacterial clearance by hemocytes (Stuart & Ezekowitz, 2008; Fauvarque & Williams, 2011), including the PDGF/VEGF receptor ortholog *Pvr* (Wood *et al*., 2006), the pattern recognition receptor PGRP-SC1/*picky* (Garver *et al*., 2006), cell-surface proteins encoded by *nimrod* (Kurucz *et al*., 2007) and *eater* (Kocks *et al*., 2005), and vesicular transport and fusion regulators, such as *psidin* (Brennan *et al*., 2007) and *full-of-bacteria* (*fob*) (Akbar *et al*., 2011). Gene expression and tissue-specific knockdown studies implicate many other molecular players [e.g. see (Ramet *et al*., 2002; Zettervall *et al*., 2004; Cronin *et al*., 2009; Zanet *et al*., 2009; Kadandale *et al*., 2010)].

Phagocytosis has been characterized primarily in cell culture or early developmental stages with little work focused on hemocytes in aging adult flies. It has been reported that new phagocytic cells are produced only during the early phases of the life cycle, and not in adult Drosophila (Lemaitre & Hoffmann, 2007), suggesting that the effects of age on these cells can be estimated with precision. Hemocyte activity in young adult flies has been tested using fluorescent bacteria, providing gross estimates of phagocytic abilities (Elrod-Erickson *et al*., 2000; Kocks *et al*., 2005; Garver *et al*., 2006). However, this whole-fly measurement is unlikely to be sensitive enough to detect small changes that might occur with age or within natural populations. Furthermore, this type of assay cannot distinguish the efficiency of phagocytic function from individual variation in hemocyte size, number, or location. An alternative method examined the phagocytic character of cells from adults, but only included nonadherent cells at one time point postinfection (Kocks *et al*., 2005; Mackenzie *et al*., 2011). Such work suggested that the number and proportion of phagocytically active cells declines with age (Mackenzie *et al*., 2011). Interestingly, studies on human neutrophils and macrophages reveal declines in phagocytic ability with age, although responses are variable depending on the cell types and assays (Plowden *et al*., 2004; Kovacs *et al*., 2009). Mechanisms underlying age-related changes in immune function remain unclear.

As phagocytosis acts as a first line of defense against pathogens after infection, we examined age-related changes in this process. Here, we evaluate the effect of age on heart-associated hemocytes and explore the aspects through which the detrimental effects of age could act on phagocytosis. We focused on heart-associated cells because many hemocytes are concentrated at this tissue (called the dorsal vessel) in adult flies (Elrod-Erickson *et al*., 2000), and recent work in mosquitoes showed preferential adhesion of hemocytes near the heart after pathogen injection (King & Hillyer, 2012).

To evaluate the age-specific immunological function of hemocytes *in vivo*, we developed and employed a quantitative phagocytosis assay. Use of *Hemese*-Gal4-driven green fluorescent protein (GFP), which specifically marks hemocytes (Kurucz *et al*., 2003; Zettervall *et al*., 2004), demonstrated that these cells are the predominant phagocytic cells in young and old flies. Knockdown of *nimrod C1* or *eater* in heart-associated adult blood cells reduced phagocytosis to a similar extent as in larvae (Kocks *et al*., 2005; Kurucz *et al*., 2007). We also found that *Rab5* promotes phagocytosis after engulfment in adults, similar to observations in cell culture (Agaisse *et al*., 2005; Cheng *et al*., 2005; Philips *et al*., 2005; Peltan *et al*., 2012).

We found a consistent decline in phagocytic efficiency with age. At older ages, we observed fewer heart-associated hemocytes. The rate of phagocytic uptake and the proportion of active hemocytes were similar in young and old flies. However, clearance of bacteria from the cell once engulfed decreased dramatically with age. This suggests that a decline in phagocytic efficiency contributes to overall immunosenescence. These observations, and a new quantitative assay for measuring phagocytic ability in adults, show that adult Drosophila can provide a powerful system for study of the genetic basis of age-related changes in immune response.

## Results

### A whole-fly assay did not reveal changes in immune function with age

Previous work using inbred Drosophila lines derived from a natural population showed significant variation in the ability of different genotypes to clear a bacterial infection at different ages (Felix *et al*., 2012). To test whether these disparities arose from altered phagocytic ability, we examined two lines from the Felix *et al*. study (numbers 387 and 437) that behaved differently in the clearance assay. Using a method described previously (Elrod-Erickson *et al*., 2000), we injected adult, virgin female flies with fluorescent *Escherichia coli* and assayed fluorescence through the cuticle. Although flies from line 387 contained more fluorescence than those from line 437 at 1 week of age (1762 ± 133 SEM (one standard error of the mean) vs. 1415 ± 52 SEM in pixel intensity measurements, *F*_1,35_ = 23.0, *P* < 0.0001), we saw no significant difference between young and old flies in either of the lines (*F*_1,35_ = 0.19, *P* = 0.7; Table 1). However, this procedure measures global phagocytosis and does not provide estimates of phagocytic events in individual hemocytes. Differences in blood cell numbers, body size, or pigmentation with age could confound the results. Thus, we sought an alternative way to measure phagocytic efficiency.

**Table 1:** Fluorescent intensity measured through the cuticle of females infected with fluorescent *Escherichia coli* shows differences by genotype but not age

Line	Number of flies	Age (weeks)		Whole abdomen fluorescent intensity	Intensity in region around dorsal vessel
387	*n* = 7	1	Mean	1762.0	1991.9
			SEM	133.0	154.0
387	*n* = 10	5	Mean	1736.4	2062.5
			SEM	90.0	138.0
437	*n* = 10	1	Mean	1415.5	1487.3
			SEM	52.0	52.0
437	*n* = 12	5	Mean	1376.7	1408.4
			SEM	27.0	32.0

### Heart-associated hemocyte numbers decline with age but remain localized

**Figure 1:**
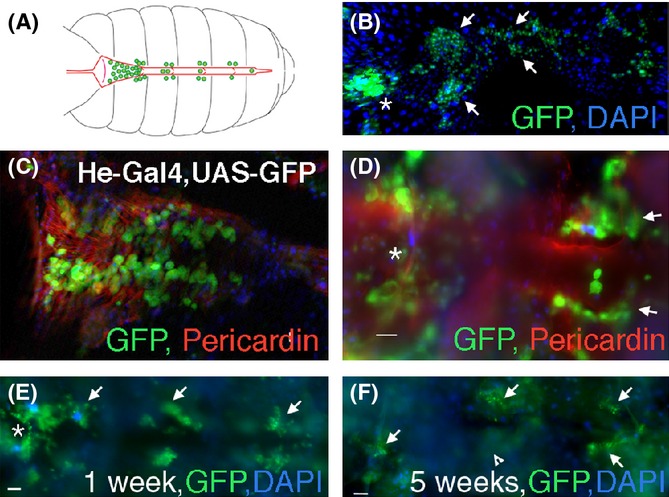
Heart-associated hemocytes decline with age but not infection. (A) A schematic of the abdominal dorsal vessel (red) and associated blood cells (hemocytes, green). Hemocytes are enlarged for clarity. Anterior is to the left in this and subsequent panels. Many circulating hemocytes cluster in the first aortic chamber of the dorsal vessel (left), while some can be seen in the other chambers. Adherent hemocytes localize just outside the dorsal vessel and along the body wall. (B) Projection of multiple optical sections of a dissected dorsal vessel and associated blood cells from a 1-week-old female, marked by GFP (green) driven by *Hemese*-Gal4 (He-Gal4). Many circulating hemocytes are seen inside the first chamber (asterisk), and clusters of sessile hemocytes are found associated laterally along the dorsal vessel. Arrows indicate clusters of hemocytes along the second and third chambers. DAPI labels all nuclei in blue. (C) An optical section of a dissected dorsal vessel from a 1-week-old female shows GFP-positive hemocytes (green, arrow) inside of the first chamber of the dorsal vessel, which is marked by staining with an antibody directed against Pericardin (red). (D) In a different optical section, heart-associated hemocytes are observed adhering outside of the first chamber (asterisk), and laterally next to the second chamber of the dorsal vessel (arrows). Two blood cells are seen in the heart. (E) A dorsal vessel from a 1-week-old He-Gal4, UAS-GFP female injected with *Escherichia coli* displays hemocytes inside the first chamber (asterisk), and clusters of hemocytes laterally along the dorsal vessel (arrows). (F) A dorsal vessel from a 5-week-old He-Gal4, UAS-GFP female injected with *E. coli* displays similar localization of hemocytes inside the first chamber (asterisk), and laterally along the dorsal vessel (arrows), although there are fewer blood cells. Arrowhead indicates an out-of-focus cluster of cells. All scale bars = 20 μm.

To observe the blood cells in greater detail, we dissected out the dorsal vessel (the fly heart) and adjacent body wall, where many hemocytes localize (Elrod-Erickson *et al*., 2000). Both circulating hemocytes, which flow with the pumped hemolymph through the heart, and sessile ones, which adhere to tissues, are fluorescently labeled in *Hemese*-Gal4 (*He*-Gal4), UAS-GFP flies (Kurucz *et al*., 2003; Zettervall *et al*., 2004). We dissected these flies to reveal the adherent hemocytes, and then stained the tissues. To identify the dorsal vessel, we added fluorescently tagged phalloidin to mark actin, or stained with an antibody recognizing the heart-enriched protein Pericardin (Fig. 1A–B) (Chartier *et al*., 2002; Demerec, 2008). We observed many hemocytes scattered individually along the dorsal abdominal wall. Some were trapped within the dorsal vessel itself, particularly in the first aortic chamber (Fig. 1A–C), and were presumed to be circulatory prior to fixation. Other hemocytes appeared in large clusters segmentally along the dorsal vessel (Fig. 1A, B, D). These clusters that adhere to tissues neighboring the hemocoel are called sessile hemocytes.

A prior study demonstrated that the number of circulating hemocytes declines with age in mated females (Mackenzie *et al*., 2011), but did not analyze the sessile cells. We examined this population in young and old *He*-Gal4, UAS-GFP virgin female flies. GFP-positive cells clustered at the abdominal dorsal vessel in both 1-week- and 5-week-old flies, but older flies possessed significantly fewer GFP-positive hemocytes than young ones (mean = 145 ± 16 SEM cells in young, *n* = 6 flies; 48 ± 7 SEM in old flies, *n* = 8; *F*_1,23_ = 30.6, *P* < 0.0001). In the same experiment, we examined the effect of infection on heart-associated hemocytes. A recent study in mosquitoes showed that infection status influences blood cell association with the dorsal vessel (King & Hillyer, 2012). Thus, we injected young and old flies with heat-killed *E. coli*, and at 90-min postinfection, compared the results with age-matched, uninfected flies. We found no significant effect of infection on the numbers of GFP-positive hemocytes localized to the dorsal vessel at 1 week of age (*F*_1,9_ = 0.09, *P* = 0.78 infected mean = 140.5 ± 20 SEM, *n* = 6) or at 5 weeks (*F*_1,12_ = 1.13, *P* = 0.30, infected mean = 54 ± 8 SEM, *n* = 8).

### A quantitative phagocytosis assay in adult flies

**Figure 2:**
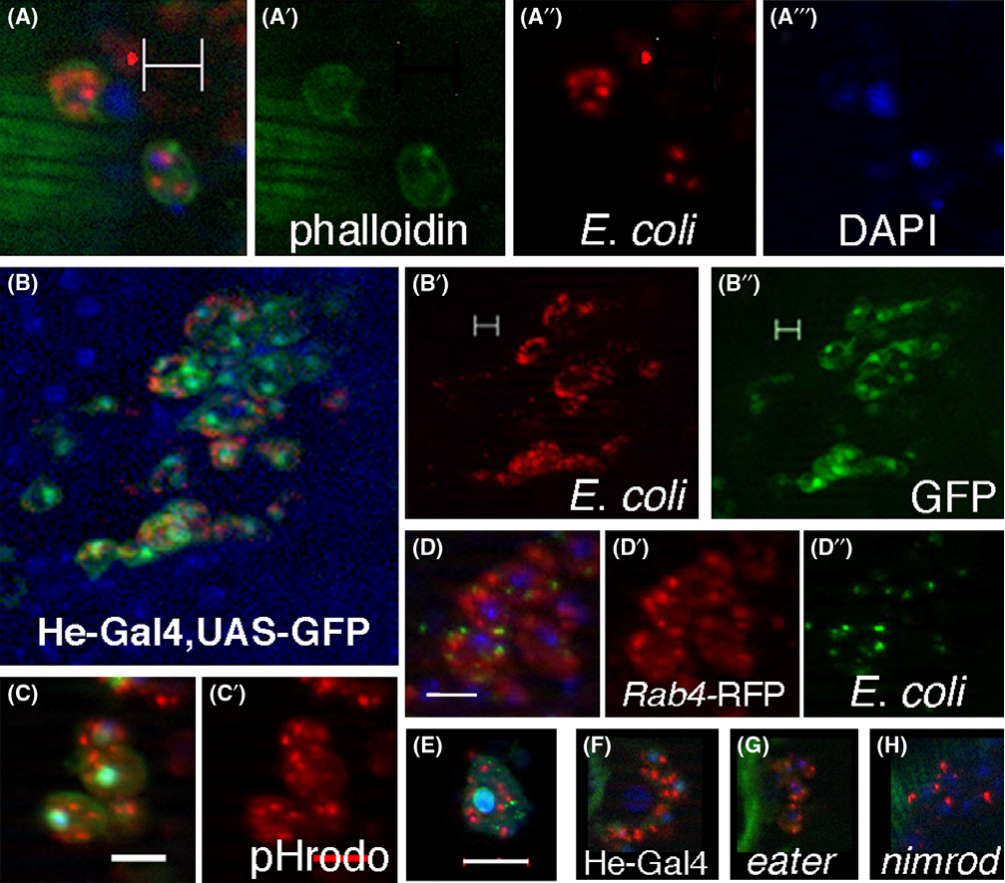
A quantitative *in vivo* phagocytosis assay. (A) Hemocytes at the dorsal vessel from an adult Canton-S female contain fluorescent *Escherichia coli* 90 min after an infection. Scale bar in all panels = 10 μm. In an optical section, Oregon Green Phalloidin staining (green, A’) indicates cortical actin; rhodamine reveals bacteria (red, A’’); and DAPI staining shows the nuclei (blue A’’’). (B) Engulfed *E. coli* (red, B’) in the dissected dorsal vessel associate with blood cells, as marked by GFP (green, B’’) via He-Gal4. (C) *E. coli* associated with GFP-marked blood cells (green) have been engulfed, indicated by pHrodo-labeled *E. coli*, which fluoresce red (C’) when phagosomes fuse with acidic lysosomes. (D) Some engulfed *E. coli* (green) colocalize with He-Gal4-driven UAS-Rab4-RFP (red), which marks endocytic vesicles. (E) A circulating hemocyte, bled from an infected He-Gal4, UAS-GFP (green) female, contains *E. coli* (red) and shows similar phagocytic character as heart-associated hemocytes. (F) Five hemocytes (distinguishable by DAPI) in the dorsal vessel show numerous phagocytic events (red) from a He-Gal4, UAS-GFP female. (G) Four hemocytes each have fewer phagocytic events than controls when *eater* is knocked down by RNAi. (H) Five hemocytes each have fewer phagocytic events than controls when *nimrodC1* is knocked down by RNAi.

Next, we developed an assay to measure phagocytosis quantitatively. This assay combines the qualitative method described above and in (Elrod-Erickson *et al*., 2000) with labeling strategies previously used in cultured, larval, or circulating cells (Ramet *et al*., 2002; Kocks *et al*., 2005; Garver *et al*., 2006; Kurucz *et al*., 2007; Fauvarque & Williams, 2011; Mackenzie *et al*., 2011). We injected Canton-S virgin female flies in the abdomen with heat-killed fluorescently labeled *E. coli,* then dissected, fixed, and stained the tissues at 90 min postinjection. Unengulfed bacteria are washed away prior to fixation, so no fluorescent quenching step is required. Many dorsal vessel-associated cells contained phagocytic events, as observed through optical sectioning (Fig. 2A–B) and consistent with previous assays. Phagocytic cells, outlined by phalloidin-stained cortical actin (Fig. 2A), consistently measured ~10 μm in diameter, the known size for hemocytes (Brehelin, 1982; Zettervall *et al*., 2004), and were particularly concentrated in and around the first aortic chamber of the heart.

To verify that hemocytes accounted for observed phagocytic events, we infected *He*-Gal4, UAS-GFP flies (Kurucz *et al*., 2003; Zettervall *et al*., 2004). We observed fluorescent bacteria overwhelmingly in association with 10-μm-diameter GFP-positive cells, many of which associated with the dorsal vessel (Fig. 2B). Optical sectioning verified that the fluorescent bacteria were within the hemocytes and not merely on the cell surface. We confirmed that the bacteria were engulfed by utilizing pH-sensitive pHrodo*-E. coli,* which only fluoresces under acidic conditions, to indicate fusion between the phagosome and lysosome. The extent of phagocytic events per hemocyte was very similar using either label (compare Fig 2A and C). Next, we injected flies expressing the early endosome marker Rab4-GFP (Yang *et al*., 2011). In many cases, fluorescent bacteria colocalized with the Rab4-GFP protein (Fig. 2D), indicating that the phagosomal cup transitioned into a vesicle. Finally, we collected circulating *He*-Gal4, GFP blood cells (Mackenzie *et al*., 2011) and found their size and phagocytic character to be qualitatively similar to those cells associated with the dorsal vessel (Fig. 2E, compare to 2A, B). These data support the idea that our phagocytosis assay specifically reflects hemocyte engulfment activity.

**Figure 3:**
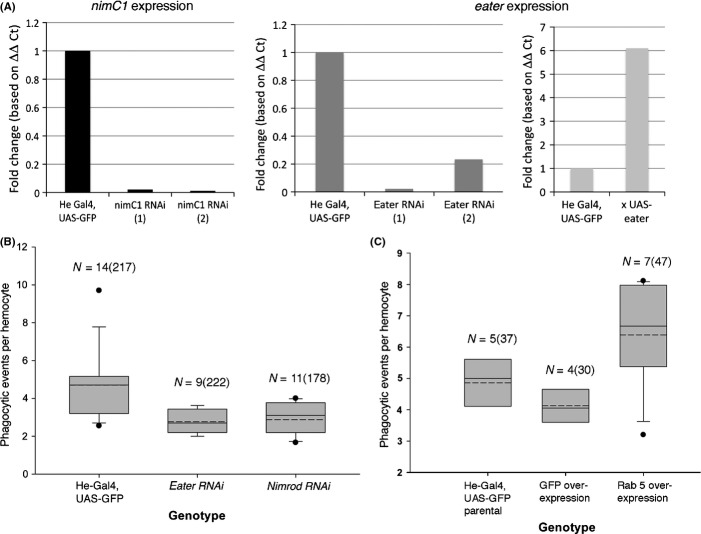
Genes required for efficient bacterial engulfment in adult Drosophila. (A) Quantitative RT–PCR confirms reduced expression of *nimC1* and *eater* transcripts in young female flies of the genotypes He-Gal4, UAS-GFP; UAS-RNAi *nimC1* (left) or *eater* (middle). Two biological replicates are shown. *He*-Gal4; UAS-GFP, UAS-eater flies showed increased gene expression and served as a positive control (right). Fold changes were calculated using △△Ct values relative to the reference gene *RpL32* (*rp49*) and the control genotype He-Gal4, UAS-GFP, using multiple technical replicates for each biological replicate. (B–C) Box plots of phagocytic ability, measured as the number of fluorescent *Escherichia coli* per active hemocyte at 90 min postinjection. Boxes represent the middle two quartiles, separated by a line representing the median, and the whiskers show the 10th and 90th percentiles. Dots indicate outliers. A dashed line marks the mean value of events per cell. *N* = number of flies and (total cells) assayed. (B) Reduced function of *eater* or *nimC1* reduces bacterial uptake by adult hemocytes. The following genotypes were compared: He-Gal4, UAS-GFP, UAS-RNAi *eater* and He-Gal4, UAS-GFP, UAS-RNAi *nimC1*. The isogenic control strain (He-Gal4) showed significantly greater phagocytic ability compared with either knockdown line (*P* < 0.05). (C) Increased Rab5 increases the number of phagocytic events per hemocyte. There is a significant difference between He-Gal4 outcrossed to UAS-GFP control vs. crossed to UAS-Rab5 wt (*P* < 0.0001). The parental control strain (He-Gal4, UAS-GFP) is also shown.

To determine the cellular efficiency of phagocytosis in adult flies, we examined this process over time by counting the number of engulfed fluorescent bacteria per active hemocyte. Time course studies showed maximal and distinct phagocytic events at 90 min for *E. coli,* and the majority of GFP-positive hemocytes in *He*-Gal4, UAS-GFP adult heart tissue contained bacteria. Later time points, after 2.5 h, revealed both punctate events and diffuse fluorescence, presumably due to degradation of the bacteria. We found that GFP-positive hemocytes contained approximately 4.5 (±0.18 SEM) events per cell at 90 min postinjection (Fig. 3B). In separate experiments, we altered the concentrations of injected bacteria. Hemocytes contained similar numbers of phagocytic events when injected with either the original or one-fifth of the titers of bacteria (see below), but had too many events to count when the bacteria were five times more concentrated. This demonstrates that the initial titer was neither limiting nor elicited the maximal possible response. Overall, our assay gives high-resolution, quantitative data in the form of bacteria engulfed per hemocyte.

### Genes required for efficient phagocytosis by adult hemocytes

We tested whether genes that function in phagocytosis in larval and cell culture assays are also required for the process in adult flies. *nimrod C1 (nimC1)* is required for efficient bacterial uptake in hemocytes in larvae, and *eater* is necessary both in larval and adult blood cells (Avet-Rochex *et al*., 2005; Kocks *et al*., 2005; Kurucz *et al*., 2007). We disrupted these genes in hemocytes by RNA interference, verified gene knockdown by qRT–PCR (at least fourfold reduction, Fig. 3A), and measured phagocytic ability by counting the number of engulfed bacteria per hemocyte. While hemocytes from the control strain (*He*-Gal4, UAS-GFP alone) contained an average of 4.5 engulfment events each, cells in which *eater* or *nimC1* were knocked down had fewer (averages of 2.9 and 3.2, respectively, Fig. 2F–H, Fig. 3B). A *post hoc* Tukey’s test (Zar, 2010) verified that the control strain differed significantly from the mutant strains (*P* < 0.05). Thus, reducing *eater* or *nimC1* function in adult hemocytes results in poorer phagocytic ability per cell (Avet-Rochex *et al*., 2005; Kocks *et al*., 2005; Kurucz *et al*., 2007).

Next, we tested whether we could disrupt phagocytosis after the initial engulfment. We examined the effect of increasing Rab5, which is known to be required for the maturation of the early endosome/phagosome prior to fusion with the lysosome (Scott *et al*., 2003; Wucherpfennig *et al*., 2003; Zhang *et al*., 2007; Morrison *et al*., 2008), and can modulate engulfment of bacteria by fly cells in culture (Agaisse *et al*., 2005; Cheng *et al*., 2005; Philips *et al*., 2005; Peltan *et al*., 2012). Overexpressed Rab5 localizes to its normal subcellular structures (Zhang *et al*., 2007), can raise internalization rates, and can increase early endosomal structures (Nielsen *et al*., 1999; Wucherpfennig *et al*., 2003). When we overexpressed Rab5 in hemocytes, phagocytic events increased significantly, with the average events per cell increasing from 4.1 in the control to 6.4 events per cell in the Rab5 overexpressing strain (post hoc contrast analysis results: *t* = 2.52, df = 12, *P* < 0.05, Fig. 3C). This is consistent with the idea that hemocytes engulf bacteria more rapidly with excess Rab5, but cannot complete phagocytic turnover as quickly, leading to accumulation. These genetic experiments further support the validity of the assay.

### Phagocytic ability changes with age

We next used the adult phagocytosis assay to explore whether hemocytic function changes with age. A prior study suggested that both the number of circulating hemocytes and the proportion that were phagocytic decline with age in adult female, mated flies (Mackenzie *et al*., 2011). We determined whether the fraction of phagocytic cells differed between young and old, virgin, female *He*-Gal4, UAS-GFP flies, focusing on the cells localized to the heart. For these experiments, we used either our regular titer of bacteria or one-fiftieth of this concentration. We found no significant difference in the proportion of phagocytic cells across age at either titer (Table 2). Ninety minutes after infection, 61 or 72% of GFP-positive cells at the dorsal vessel in 1-week-old flies contained engulfed bacteria at low and regular titers, respectively. For 5-week-old flies, 64 or 85% of the cells were phagocytically active at low and regular titers, respectively (Table 2). Interestingly, the mean percentage of phagocytically active cells was higher at both ages when more bacteria were injected. Thus, the proportion of hemocytes associated with the dorsal vessel that are phagocytic does not decline with age.

**Table 2:** No significant difference in proportion of phagocytically active hemocytes at 1 and 5 weeks of age

Titer	Age	Number of flies and (number of GFP+ cells) analyzed	% Active	% Inactive
Low	1 week	*n* = 5 (153 cells)	61	39
Low	5 weeks	*n* = 4 (103 cells)	64	36
Regular	1 week	*n* = 16 (253 cells)	72	28
Regular	5 weeks	*n* = 11 (245 cells)	85	15

*He*-Gal4, UAS-GFP flies injected with fluorescent *E. coli* assayed ninety min after infection. If GFP-positive cells had at least one phagocytic event, they were counted as active; GFP-positive cells with no events were classified as inactive. Only GFP-positive cells associated with the dorsal vessel were scored. We analyzed the data using a maximum likelihood analysis with nested logistic regression and a binomial distribution and detected no significant difference in active proportion with age: low titer (1:50th): χ^2^ = 0.001, *P* = 0.97; regular titer: χ^2^ = 1.13, *P* = 0.28. Ages given are either 1 week posteclosion (4–7 days) or 5 weeks posteclosion (35–39 days).

**Figure 4:**
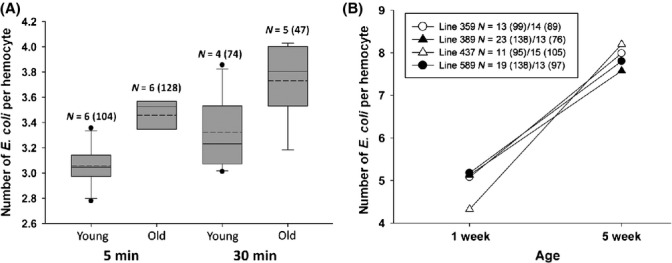
Engulfed bacteria per hemocyte increases with age. (A) Box plots of phagocytic ability, measured as the number of fluorescent *Escherichia coli* per active hemocyte in young (5–7 days) and old (35–39 days) He-Gal4, UAS-GFP flies, injected with low concentrations of *E. coli*. Dashed lines indicate the means. (See text for statistical analysis.) (B) Results of the phagocytosis assay for four natural variant lines at one and 5 weeks of age. While all four lines show a significantly higher number of bacteria per active phagocytic cell with age (see text), genotypes differ slightly in the way that age affects this trait. Values shown are averages for each genotype at 1 and 5 weeks, respectively; *N* = number of flies and (number of cells) assayed at 1 and 5 weeks.

To look at phagocytosis on a cellular level, we assayed engulfment events per cell in young and old flies over time in our low-titer experiment. At this concentration, not all cells with the potential to be phagocytic are active (Table 2). However, among active cells, we found a significant effect of age on initial uptake of bacteria and later numbers of engulfment events. We observed an average of 2.09 and 2.62 engulfed bacteria per cell at 5 min postinjection in 1-week- and 5-week-old flies, respectively (Fig. 4A, *F*_1,163_ = 6.16, *P* = 0.01). 30 min after a low-titer infection, hemocytes from older flies contained 3.01 events compared with 2.21 events per cell in younger flies (Fig. 4A, *F*_1,226_ = 4.90, *P* = 0.03). Thus, we saw on average fewer events per cell at low titer than regular titer (compare to Fig. 3B–C). However, there were more events per active cell in older individuals at either time point.

Prior work in twenty inbred fly lines derived from a natural population found extensive genetic variation in the ability to clear an *E. coli* infection at different ages (Felix *et al*., 2012). Thus, we wanted to determine whether phagocytic ability specifically varied among lines and how it was influenced by age. It was possible that the increase in engulfment events per active cell was due to fewer hemocytes in older flies. Thus, we returned to our normal injection concentration (1 × 10^6^ *E. coli* per μL), which provides an excess of *E. coli* per hemocyte, and assayed natural variant strains. We chose four lines to assay: two of these exhibited immunosenescence in the prior clearance assay, but the other two had improved clearance ability at 4 weeks of age (Felix *et al*., 2012).

All four natural variant lines revealed a strong, consistent effect of age on phagocytosis. Hemocytes in virgin female flies from the four lines had strikingly similar numbers of phagocytic events and displayed a significant difference in the number of engulfed bacteria between young and older ages (*F*_1,113_ = 43.7, *P* < 0.0001, Fig. 4B). Surprisingly, in all lines, hemocytes of the 5-week-old flies contained more engulfed bacteria (an average of 7.9 phagocytic events/cell) than the hemocytes of the 1-week-old flies (5.0 events/cell; Fig. 4B).

Several ideas, none mutually exclusive, could explain why older cells had more phagocytic events in these assays. One is that fewer hemocytes in old flies result in an effectively higher concentration of bacteria to engulf per cell. While this likely accounts for some differences, especially at low titers, the data when *E. coli* is in excess suggest this is not the only explanation. A second possibility is that older cells have improved phagocytic ability compared with younger flies and engulfed more bacteria. A third alternative is that hemocytes from younger flies were able to clear the bacteria by completing phagocytosis, whereas completion of the process was delayed in older flies, leading to an accumulation of bacteria in the blood cells of older flies.

### Completion of phagocytosis slows in older flies

**Figure 5:**
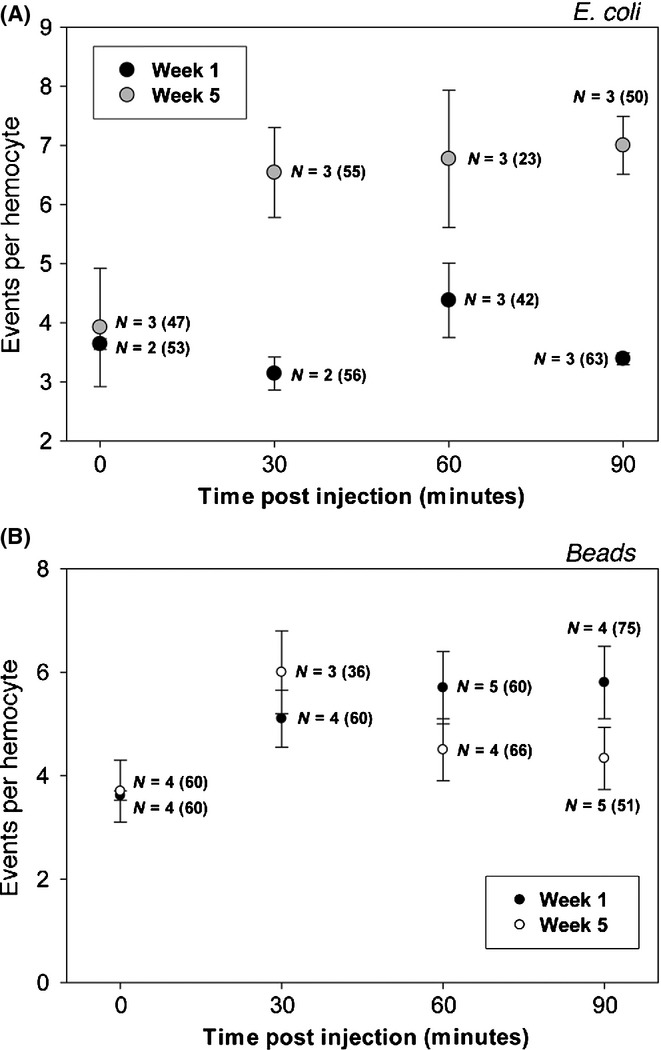
Phagocytic events accumulate in older flies. (A) One- and five-week-old flies from the natural variant line 387 were injected with fluorescent *Escherichia coli* and dissected at indicated times. Phagocytic events were counted for active cells only. Young flies exhibit a near-constant number of engulfed bacteria, while older flies accumulate bacteria with time. *N* = number of flies and (total cells) assayed. (B) Time course of bead engulfment. One- and five-week-old flies from the natural variant line 387 were injected with fluorescently labeled plastic beads, which are not broken down by phagocytosis. Flies were dissected and fixed at indicated times. Hemocytes of young and old flies appeared to engulf and accumulate beads at a very similar rate, with no statistically significant difference between the ages. (See text for statistical analyses).

If phagocytic processing differed in young and old flies, it was possible that a more detailed time course would provide insight on these differences. Thus, we measured phagocytic ability at 1 and 5 weeks of age for one of the natural variant strains at different times postinjection. Notably, at an early time (5 min), the number of engulfment events in active cells was not significantly different (*F*_1,96_ = 3.01, *P* = 0.09) with age (Fig. 5A). This further supports that initial phagocytic uptake is not adversely affected by age and demonstrates that immune-challenged hemocytes function rapidly and similarly in young and old flies. However, data from the remaining time points (with one exception at 60 min where we had fewer observations) showed significantly more engulfed bacteria in the hemocytes of 5-week-old flies (Fig. 5A, 30 mins: *F*_1,107_ = 3.01, 45.12, *P* < 0.0001; 60 min: *F*_1,61_ = 0.34, *P* = 0.56; 90 min: *F*_1,109_ = 39.37, *P* < 0.0001). The apparently constant number of phagocytic events in younger cells over the time course could be due to the combination of degradation of older phagosomes and new events. Thus, the increase in phagocytic events over time in older cells suggested that there is an accumulation of phagosomes in older individuals due to a lack of degradation/turnover.

### Phagocytic turnover declines with age

To test the hypothesis that the higher number of phagocytic events in old blood cells was due to their inability to destroy bacteria efficiently, we employed a bead engulfment assay (Elrod-Erickson *et al*., 2000; Stroschein-Stevenson *et al*., 2006). The efficiency with which hemocytes engulf the beads depends on bead size, but is similar to microbial uptake (Elrod-Erickson *et al*., 2000; Mackenzie *et al*., 2011). Unlike bacteria, the fluorescent beads are not destroyed by the cell, and instead accumulate. We injected 1-μm fluorescent beads into flies of the natural variant strain at 1 and 5 weeks of age, and examined blood cells associated with the dorsal vessel at 5, 30, 60, and 90 min. At 5 min, the number of engulfed beads was similar to the number of events when *E. coli* was used (compare Fig. 5A and B). We found that the number of engulfed beads was not significantly different between cells from young and old flies at any time point (Fig. 5B, 5 mins: *F*_1,116_ = 1.28, *P* = 0.26; 30 min: *F*_1,92_ = 0.01, *P* = 0.92; 60 min: *F*_1,125_ = 2.24, *P* = 0.14; 90 min: *F*_1,122_ = 0.68, *P* = 0.41), suggesting uptake occurs at a similar rate. Moreover, this indicates the change in cell number with age does not affect initial uptake. This suggests that old and young hemocytes were able to process engulfment events similarly, implying that the difference observed with *E. coli* was due to defective turnover in older cells.

## Discussion

Immunosenescence is a common phenomenon, but the precise contributions of immune cells and molecular pathways to this decline are not yet resolved. Drosophila provide a powerful, genetic, model system for studying this issue; however, definitive assays to measure age-related decline in immune response had not been worked out. Most prior studies were limited by their dependence on assaying indicators of immune function [e.g. (Zerofsky *et al*., 2005)] or summative aspects of immunity (Lesser *et al*., 2006; Ramsden *et al*., 2008; Felix *et al*., 2012) [but also see (Mackenzie *et al*., 2011)]. We have developed a quantitative phagocytosis assay useful for understanding the functional effects of age on immune functions and for studying the genetic basis of age-related changes in the efficacy of phagocytosis. We show here that our method specifically measures phagocytosis by blood cells and that adult fly blood cells require several of the same genes as hemocytes in larvae and other cell types for efficient phagocytic processing.

As was previously described (Mackenzie *et al*., 2011), we observed a decline in hemocyte number and function with age. However, our results do not entirely match those of Mackenzie *et al.,* and several possibilities could explain these differences. One discrepancy was that we did not detect a significant decline in the proportion of cells that were phagocytically active with age. This could be due to variation in the assays, including the use of different genotypes, differences in mating status (our study used virgin instead of mated flies), varied infection titers and measurement time points, or more subtle differences in growth conditions. Moreover, our work focused on heart-associated cells, which may behave differently than the circulating cells characterized before. Indeed, in mosquitoes, phagocytic cells preferentially associate with the heart upon infection (King & Hillyer, 2012). In any case, fewer phagocytic cells at old age can contribute to immunosenescence by increasing the bacterial load on each hemocyte. Additionally, our data show that the completion of phagocytosis begins to fail on a cellular level.

While one could imagine that all cellular processes would deteriorate with age, we found this not to be the case. In particular, the proportion of heart-associated hemocytes engaged in phagocytosis and the rate of initial bacterial uptake were similar at either age. Instead, there appeared to be a decline in processing phagocytic vesicles. One possibility is that this phenotype results from a decrease in vesicle/membrane availability. Such a defect may arise due to changes in autophagy, which is responsible for normally destroying defective organelles and molecular debris, and has been strongly associated with aging in many organisms (Melendez & Neufeld, 2008; Partridge, 2008; Young & Narita, 2010). Additional work is necessary to test this hypothesis.

Natural variant lines exhibit different abilities to clear infections with age (Felix *et al*., 2012). We tested four lines in our assay—two with improved bacterial clearance at older age and two having worse clearance with age (Felix *et al*., 2012). We postulated these lines would have different phagocytic abilities, but they did not. Instead, the general effect of age on phagocytosis was the same among all genotypes tested: All lines showed a significant increase in the number of phagocytic events per hemocyte in older flies. Mackenzie *et al*. (2011) estimated that the number of hemocytes in old individuals was 90–66% of that in young flies, which contributes to an increase in bacteria per blood cell. However, given that our injected *E. coli* titers were not limiting at either age and that more cells are active at higher infection levels, we do not believe that fewer hemocytes sufficiently explains why there are more events per cell. Instead, we propose the difference with age is largely due to a decrease in the rate of destruction of engulfed bacteria. An accumulation of phagocytic events in older flies may explain why the change with age was not detected when we used the assay by Elrod-Erickson *et al*. (2000)—fewer hemocytes with more events in old flies looked similar to more blood cells with fewer events each in young animals. Fewer cells combined with poorer phagocytic processing likely gives rise to an overall poorer immune response with age. Mechanistically, the differences in bacterial clearance observed in the natural variant lines reported by Felix *et al*. (2012) must be due to a combination of both arms of the immune response, and future studies should address these components simultaneously.

A detailed characterization of the ability of hemocytes to engulf and degrade bacteria is important in understanding the cellular aspects of the innate immune response. We have shown a potential mechanism that contributes to immunosenescence, above and beyond the loss of blood cells with age. Much remains to be done to parse the relative influence of phagocytosis and AMP production on immune function with age. The combination of the quantitative phagocytosis assay and extensive genetic resources in Drosophila will enable us to determine key components contributing to immunosenescence and to implicate molecules that may have conserved functions in humans.

## Experimental procedures

### *In vivo* phagocytosis assay

Flies were injected into the abdomen with heat-killed fluorescent *E. coli* (Bioparticles, tetrarhodamine, Alexa Fluor 488-, or Alexa Fluor 594-conjugated, or pHrodo (E-2862, E-13231, E-23370, P35361). Invitrogen/Life Technologies, Carlsbad, CA) using an Eppendorf FemptoJet microinjector set for the following conditions: The FemptoJet parameters were pi [PSI] = 0.46, ti[s] = 0.2, pc [PSI] = 0.20. The stock solution contained 6 × 10^9^ *E. coli* particles per mL, which was diluted 1:6 for injections, unless otherwise indicated. For bead assays, we used Alexa Fluor 568-labeled 1-μm beads (Invitrogen, F-13083) at the same concentration. For natural variant comparisons, injections were always carried out in the morning to avoid changes in immune function due to circadian rhythms [e.g. as observed in (Lazzaro *et al*., 2004)]. Postinjection, flies recovered at room temperature for 90 min or the times indicated prior to dissection and fixation.

The dorsal vessel and attached abdominal cuticle of each fly were dissected in Schneider’s Media (Invitrogen) supplemented with 15% fetal bovine serum and 0.6 × Pen/strep, fixed in 4% formaldehyde (MeOH-free, Electron Microscopy Sciences, Hatfield, PA) for 15 min, and washed in PBS + 0.1% Tween. For cortical actin staining, we used Oregon Green 488 Phalloidin (1:8, Invitrogen O-7466) in PBS. Abdomens were mounted on slides dorsal side down in 70% glycerol. In trials with the natural variant line 397, we did not use Phalloidin staining. In these assays, we counted only bacteria within a 10-μm-diameter circle centered on the cell nucleus.

Images were acquired using the Zeiss AxioImager.Z1 fluorescent microscope (Zeiss, Jena, Germany). The ApoTome (Zeiss, Germany) structural interference system was used generate optical sections; images were acquired and analyzed with AxioVision software (Zeiss, Munich, Germany). Images were oriented and formatted in Adobe Photoshop and Adobe Acrobat (Adobe, San Jose, CA, USA).

### Genetic strains

As *Hemese* (*He*) expression is confined to blood cells, we used *He*-Gal4 driving GFP (w^*^; P{w[+mC] = *He-Gal4*.Z}85 or w^*^; P{w[+mC] = *He-Gal4*.Z}85, P{w[+mC] = *UAS-GFP.nls*}8 (Zettervall *et al*., 2004). For the Rab4-RFP assay, we crossed *He-Gal4* virgin females to w[*]; P{w[+mC] = UAS-Rab4-mRFP}2 (Yang *et al*., 2011) (flybase.org). For knockdown of *eater* and *nimrodC1*, *He-*Gal4, UAS-GFP females were crossed to *y*^*1*^*, v*^*1*^; P{y[+t7.7] v[+t1.8] = TRiP.JF01884}attP2 or *y*^*1*^*, v*^*1*^; P{y[+t7.7] *v*[+t1.8]=TRiP.JF01793}attP2, (Ni *et al*., 2008), respectively, or to UAS-eater (Kocks *et al*., 2005) for a qRT–PCR control. For Rab5 experiments, *He-*Gal4*,* UAS-GFP virgin females were crossed to y[1] w[*]; P{w[+mC] = UASp-YFP.Rab5}Pde8[08b] (Zhang *et al*., 2007). Stocks listed above were provided by the Bloomington Stock Center, Bloomington, IN. The four inbred lines derived from the natural population of Drosophila in Raleigh, NC, (359, 387, 437, and 589), were kindly provided by the laboratory of Trudy Mackay at North Carolina State University (Mackay *et al*., 2012).

### Quantitative RT–PCR

Total RNA was isolated from *He*-Gal4, UAS-GFP, and *He*-Gal4, UAS-GFP; TRiP UAS-RNAi *nimC1*, and *He*-Gal4, UAS-GFP; TRiP UAS-RNAi *eater*, and *He*-Gal4, UAS-GFP; UAS-*eater* females using the RNeasy Isolation Kit (Qiagen, Germantown, MD, USA). cDNA synthesis from total RNA was performed using the iScript cDNA Synthesis kit (BioRad, Hercules, CA, USA), and potential genomic DNA contamination was removed using Turbo DNA-free (Ambion; Life Technologies, Carlsbad, CA, USA). Quantitative RT–PCR was performed with iTaq Universal SYBR-Green Supermix (BioRad) with a BioRad iCycler iQ system. The following primer pairs were used: *eater*: 5′-GGAAGTGGCTTCTGCACGAAAC-3′ and 5′-CGACTACATCCCTTGCAGTAGGG-3′ *nimC1* (RA) 5′ GTTTGTAACCGATCGCAGGTGG and 5′-TCGTAGCCCTCACAGCAACTG-3′ *RpL32 (rp49):* 5′-GTGAAGAAGCGCACCAAGCAC-3′ and 5′-ACGCACTCTGTTGTCGATACCC-3′. Serial dilutions confirmed the amplification rates, and delta Ct analysis was carried out relative to RpL32 gene expression. At least two sets of five flies from each genotype were assayed, with multiple technical replicates.

### Statistical analysis

To account for possible small variation in injection volumes between experiments, each experiment was completed on a single day and repeated. We calculated phagocytic event data using multiple individuals per genotype at each age as indicated, and at least 10 blood cells from each individual. Box and whisker plots illustrate the ranges of numbers of phagocytic events. Differences in phagocytic events were analyzed with either fixed-effects ANOVA or mixed-model nested ANOVA [Proc Mixed in SAS V9.2, (Zar, 2010)]. Mixed models were used to test the main fixed effects of age and genotype and the random effect of individuals nested within genotype and age (whenever multiple blood cell counts were used per individual in the phagocytosis assays). We used independent contrasts (Judd & McClelland, 1989) in post hoc tests following mixed-model analyses to test for significant differences among targeted genotypes. Blood cell counts were transformed to √*x +* √*x + 1* to satisfy assumptions of ANOVA (Snedecor & Cochran, 1989). We used maximum likelihood to estimate the effects of age on the proportion of blood cells that had engulfed bacteria using a nested logistic regression model with individuals nested within age [implemented by Proc Genmod in SAS V9.2, (Allison, 1999)].

### Antibodies

Antibody staining and DAPI staining (1:1000, Invitrogen/Life Technologies, D1306) were executed using standard procedures. The following antibodies were used: rabbit anti-GFP (1:1000) (Invitrogen, A6455), Alexa Fluor 488 anti-rabbit (1:400) (Invitrogen, A11008), mouse monoclonal antibody directed against Pericardin, EC11 (1:5) (Chartier *et al*., 2002) (Developmental Studies Hybridoma Bank), Alexa Fluor 568 anti-mouse (1:400) (Invitrogen, A11031).
